# Sputum smears quality inspection using an ensemble feature extraction approach

**DOI:** 10.3389/fpubh.2022.1032467

**Published:** 2023-01-25

**Authors:** Amarech Kiflie, Guta Tesema Tufa, Ayodeji Olalekan Salau

**Affiliations:** ^1^Faculty of Electrical and Computer Engineering, Arba Minch Institute of Technology, Arba Minch, Ethiopia; ^2^Department of Electrical/Electronics and Computer Engineering, Afe Babalola University, Ado Ekiti, Nigeria; ^3^Saveetha School of Engineering, Saveetha Institute of Medical and Technical Sciences, Chennai, Tamil Nadu, India

**Keywords:** GLCM, CNN, KNN, sputum smear inspection, feature extraction

## Abstract

The diagnosis of tuberculosis (TB) is extremely important. Sputum smear microscopy is thought to be the best method available in terms of accessibility and ease of use in resource-constrained countries. In this paper, research was conducted to evaluate the effectiveness of tuberculosis diagnosis by examining, among other things, the underlying causes of sputum smear quality for Ethiopian states such as Tigray, Amahira, and Oromia. However, because it is done manually, it has its limitations. This study proposes a model for sputum smear quality inspection using an ensemble feature extraction approach. The dataset used was recorded and labeled by experts in a regional lab in Bahir Dar, near Felege Hiwot Hospital after being collected from Gabi Hospital, Felege Hiwot Hospital, Adit Clinic and Gondar Hospital, as well as Kidanemihret Clinic in Gondar. We used a controlled environment to reduce environmental influences and eliminate variation. All the data was collected using a smartphone (the standard 15) with a jpg file extension and a pixel resolution of 1,728 × 3,840. Prior to feature extraction, bicubic resizing, and ROI extraction using thresholding was performed. In addition, sequential Gaussian and Gabor filters were used for noise reduction, augmentation, and CLAHE was used for enhancement. For feature extraction, GLCM from the gray label and CNN from the color image were both chosen. Ultimately, when CNN, SVM, and KNN classifiers were used to test both CNN and GLCM features, KNN outperformed them all with scores of 87, 93, and 94% for GLCM, CNN, and a hybrid of CNN and GLCM, respectively. CNN with GLCM outperformed other methods by 0.7 and 0.1% for GLCM and CNN feature extractors using the same classifier, respectively. In addition, the KNN classifier with the combination of CNN and GLCM as feature extractors performed better than existing methods by 1.48%.

## 1. Introduction

Tuberculosis (TB) is a major public health problem in the world. It is a bacterial infection caused by Mycobacterium tuberculosis. In 2018, over 10 million people worldwide were diagnosed with tuberculosis (TB) (1). TB is a communicable disease which spreads rapidly from affected patients to healthy patients. Most of the time, it largely affects the lungs of the body, but it can damage the spine, kidney, brain, and other parts of the human body. The basic causes of this disease are: (i) contact with a person that has active TB, lacking adequate healthcare, and (ii) when healthcare workers and individuals live in substandard conditions and use untreated medical equipments. The signs of active TB are coughs within 3 weeks or more, chest pain, coughing up blood, feeling tired all the time, night sweats, chills, fever, loss of appetite, or weight loss (2).

A number of factors, including social disparities in health, a lack of qualified local healthcare providers, and inadequate healthcare infrastructure in resource-constrained and developing nations, pose obstacles to efforts to reduce or eradicate this communicable disease. Therefore, it is clear that creating cost-effective mechanisms is an extremely important issue for medical treatment (3). Early diagnosis, effective treatment (therapy) are important ways to reduce the rate of the spread of this communicable disease (TB). Because technology interacts with and makes life easier for people, particularly when it comes to the internet, including cloud computing, wireless sensor networks, the ubiquitous internet of things, and algorithms like artificial intelligence, machine learning, and deep learning, researchers want to automate the diagnosis of this communicable disease. A number of studies have been conducted recently to diagnose TB using automated methods with machine learning and shallow learning algorithms on raw data from chest x-rays and sputum smear microscopy (4). To diagnose TB disease, highly trained medical professionals are required to study different body's part by using chest X-ray, Skeen test, smear microscopy, culture test, and gen expert machine. Because manual diagnosis is time-consuming and costly, automated diagnosis of TB by using smear microscopy and chest X-ray is being experimented by different researchers (5, 6). The majority of studies only consider the detection and diagnosis of tuberculosis to determine whether it is positive or negative but did not consider accessibility, maintainability, quality, or any other factors. Chest x-rays or smear microscopy are almost exclusively used methods for research on automated TB system diagnosis. In terms of usability, accessibility, and maintainability, the sputum smear microscopic detection method for tuberculosis is the gold standard (3, 6, 7). To the best of the author's current knowledge, there is no research work conducted to assess sputum smears quality or Tb detection and diagnosis using CNN, SVM, and KNN classifiers. In addition, although some of the methods have been used to minimize the root cause of TB, they are mostly not automated for examination and classification.

The outcome of misdiagnosed is most times as a result of poor sputum smears preparation. This results in multi-drug resistance, ease of transmission through contact, resources are wasted and lives are lost. Additionally, external quality control (EQA) programs are needed to ensure that smears are prepared and interpreted correctly and that all laboratory centers achieve an acceptable level of performance to overcome the stated limitations. However, effective EQA programs are labor-intensive, prone to error, delay, and require dedicated staff for onsite supervisory visits, and rechecking of results for relatively large numbers of smears which is usually tedious.

In this work, we evaluated handcrafted and deep learning algorithms for sputum smear preparation. This is aimed at assisting medical professionals in examining, classifying, and diagnosing TB using sputum smear microscopy.

The following are the most important contributions of this research study:

- Enhanced pre-processing of Sputum smear quality inspection.
- Identification of appropriate feature vectors for Sputum smear quality inspection, and
- Improvement of the Sputum smear quality inspection performance.

The remaining sections of the paper are organized as follows: Section 2 presents a review of related works, Section 3 presents the proposed methodology, Section 4 presents the experimental results and discussion, and finally the paper is concluded in Section 5.

## 2. Literature review

A number of researchers have conducted research for detecting and diagnosing tuberculosis (TB). Even though there are various ways for diagnosing TB, the majority of them rely on sputum smears and chest X-rays to automate TB detection using machine learning and computer vision approaches.

Ayaz et al. (5) demonstrated how important feature extraction is to the TB model's detection success. Using a combination of handcrafted and deep learning feature extraction methods, the authors tried to solve this difficulty. The model's performance was assessed using a variety of measures. Additionally, they tried to categorize TB into various classes based on different symptoms by treating the different TB manifestations based on different degrees of severity. Finally, comparison was made with binary classifiers (normal and abnormal), and the results show that the number of classes available had a substantial effect on the model's performance because the average precision of binary classifiers is higher than multiclass classifiers.

Mithra and Emmanuel (6) used a deep learning-based approach for TB diagnosis. The dataset type, multiple data sets, and algorithm types are improved day by day and year by year, as shown in this survey work, in order to achieve superior performance and classification accuracy, sensitivity, and specificity by reducing various loss and cost functions. To evaluate the accuracy of tuberculosis diagnosis, the authors only used x-ray datasets. Another point is that while deep learning architectures like CNN, Google net, and Alex net can be used to diagnose these infectious and highly contagious diseases, only a small amount of data (up to 100) was initially used by the researchers, and features were manually extracted; however, this data was insufficient for this extensive study.

Asrat et al. (8) proposed a system which selected blinded rechecking for three external quality assessment of TB laboratory evaluation mechanisms (onsite evolution, blind rechecking, and panel testing) assessed the performance of laboratories in 81 healthcare centers. The achieved detection rate of TB was 82.7 and 81%, respectively.

Lorent et al. (9) proposed an approach which was used for offering and receiving TB awareness from door-to-door, especially in metropolitan areas. Active case finding (ACF) of tuberculosis screening, as opposed to passive case finding (PCF), is a potential method for promoting early case discovery and treatment. The results show that active case examination in the urban impoverished community of Cambodia presented difficulties in the detection and treatment of tuberculosis.

Mekonen et al. (10) identified the causes of poor sputum smear consistency for acid-fast bacilli identification in Ethiopian health facilities. By distributing questionnaires that were gathered and analyzed using SPSS software, a cross-sectional research of health center laboratories was evaluated in order to have a fundamental understanding of the current working system to identify the gap. 35.4% of the main technical errors, 23.6% of the false-negative results, and 30.9% of the false positive results within 55 TB health center laboratories were examined. Additionally, an assessments was carried out for specimen quality, smear size, smear technicians, staining, and evenness distribution. Lack of internal quality control (IQC), external quality assessment (EQA), and quality improvement (QI) of laboratory services were all discovered to be strongly related to this issue.

Saini et al. (11) presented an approach to reduce inpatient treatment delays and different infrastructural limitations such as internet and transportation. Among resource-poor disadvantaged communities, mobile apps, and deep learning health technologies were recommended to improve TB diagnosis. Their goal was to limit tuberculosis spread by treating it early and giving assistance to all in urban and rural areas.

## 3. Proposed methodology

**Figure 1:**
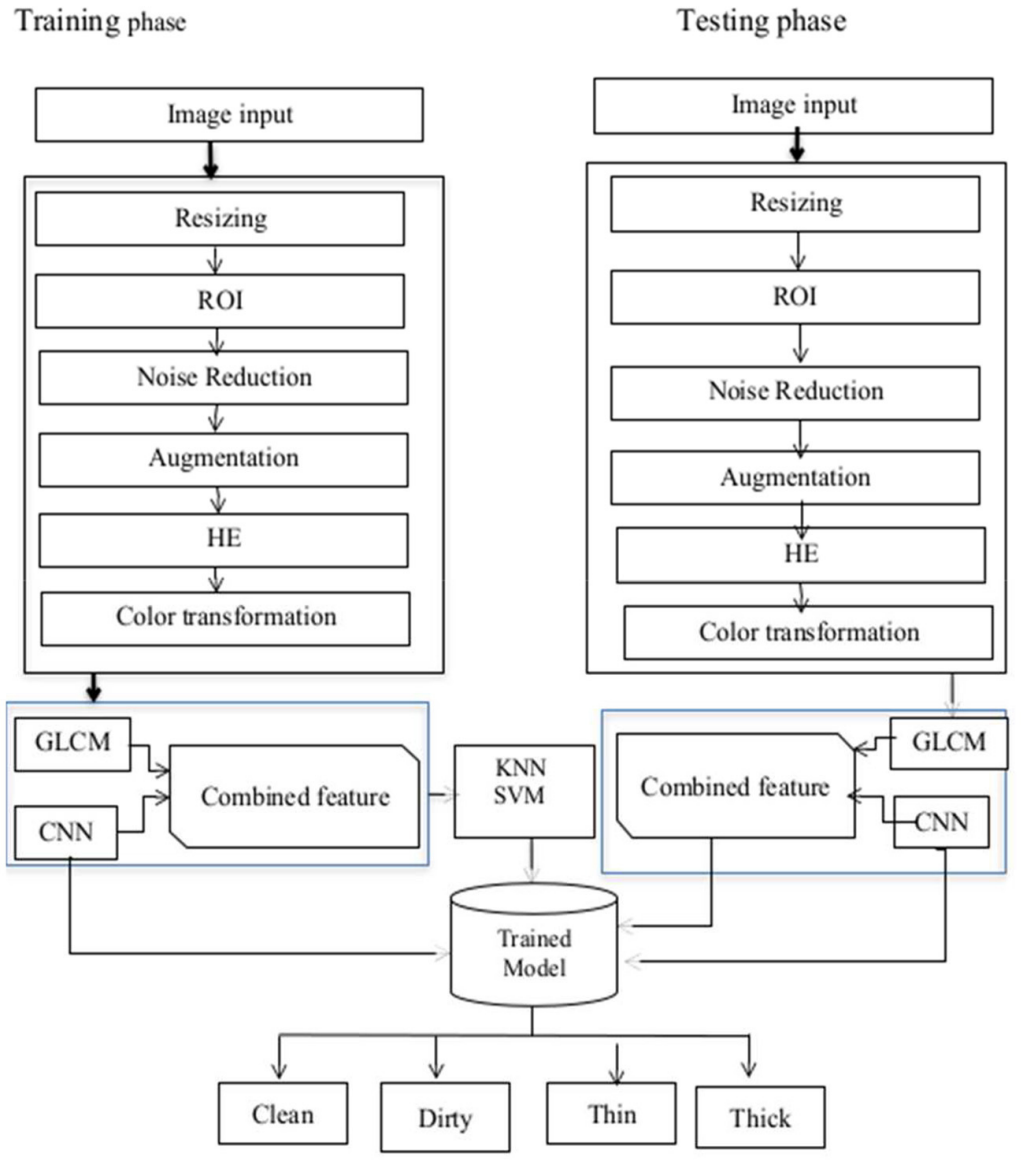
Proposed model architecture.

The proposed model comprises of four stages which are pre-processing [resizing, filtering, and histogram equalization (HE)], segmentation of the region of interest (ROI), feature extraction, and finally classification. Convolution, activation, and pooling layers are built on top of each other in the training phase to learn features and their characteristics from deep features. After features are learned, classification is performed. The proposed models architecture is shown in Figure 1.

### 3.1. Pre-processing

Pre-processing is used to improve image data by reducing undesired distortions or increasing specific visual qualities that are important for subsequent processing (12). Because geometric adjustments to images such as rotation, scaling, and translation are equivalent, they are classified as pre-processing methods.

**Figure 2:**
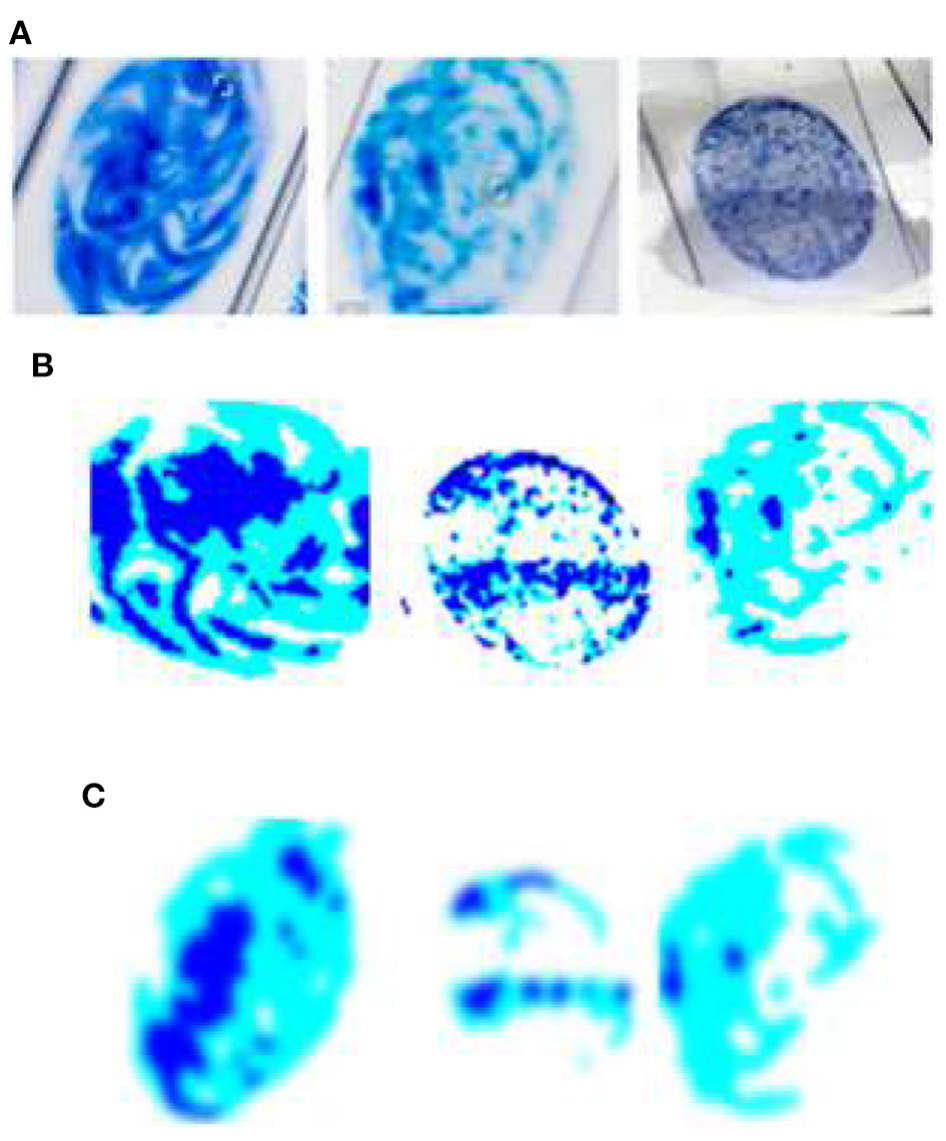
Sample of original image and the enhanced image after the application of pre-processing techniques. **(A)** Relevant data identification using ROI extraction; the most important features which aid identification are extracted. **(B)** Foreground data; extraction of the foreground of an image for further processing. **(C)** Image enhancement (IE) using Gaussian filter; IE due to random changes in intensity, lighting fluctuations, or insufficient contrast.

Figure 2 shows the sample of original image and the enhanced image after application of pre-processing techniques. In this study, we employed smartphones to collect images of sputum smears using the jpg file extension. The regional labs, Abay Mado, Adet Clinic, and Gondar Hospital, as well as Kidanemihret Clinic, used are located near in Felegehiywot Hospital, Bahir Dar Health Center, and Addisalem Health Center. We were unable to obtain enough more data because the examination was conducted quarterly and was discarded after the results were analyzed. In all, 892 images were acquired for the study. After data gathering, pre-processing techniques were used to obtain improved images and information for the next stages of segmentation and feature extraction. Furthermore, resizing, noise filtering, and histogram equalization with color transformation were performed. The acquired dataset was categorized into four classes such as thin, thick, clean, and dirty. In order to reduce the data samples in the majority class, an undersampling technique was applied to enable the minority class to have the same number of data as the majority class. To carry out undersampling, we employed the Pandas sampling technique.

#### 3.1.1. ROI extraction

Image segmentation is the split or separation of an image into areas, with each area consisting of pixels that are similar in color, intensity, or texture. The results of image segmentation are a set of segments that include the entire image. The purpose of segmentation is to break down or divide an image into several parts for subsequent study. The threshold selection criteria are the largest inter-class variance between the background and the target image (13).

- *Gaussian ROI extraction algorithm*

The algorithm for the Gaussian ROI extraction is as follows.

//Input Data: Resized image

//Output Data: foreground image

Begin:

Image = read image

Thresh = threshold (image, 100, 255, cv2.THRESH)

Return thresh image

End

#### 3.1.2. Noise filtering

Noise in images refers to undesirable information or simply undesired pixels in original digital images caused by light, color brightness, and other factors. Noise produces undesirable results such as unrealistic edges, artifacts, unseen lines, blurred objects, and corners (14). Noise fading is used to improve the data and allow for accurate pixel representation. Smear slides can reduce the visibility of noise by removing or reducing noise by smoothing the entire image and leaving areas of high contrast.

#### 3.1.3. Gaussian filter

When a camera or other imaging system takes an image, the vision system intended for it is frequently unable to immediately use it. Random changes in intensity, lighting fluctuations, or insufficient contrast may distort the image, which must be addressed in the early stages of processing (15). When the noise is Gaussian in character, the Gaussian filters work well because they're done linearly. This filter's overall effect is to blur the image, essentially removing Gaussian noise. This is illustrated in Figure 2.

- *Gaussian filter algorithm*

//Input Data: Segmented image

//Output Data: Enhanced image

Begin:

Image = read image

Blur = cv2.GaussianBlur (img1, (9, 9), channel)

Return Blur image

End

#### 3.1.4. Contrast limited adaptive histogram equalization

**Figure 3:**
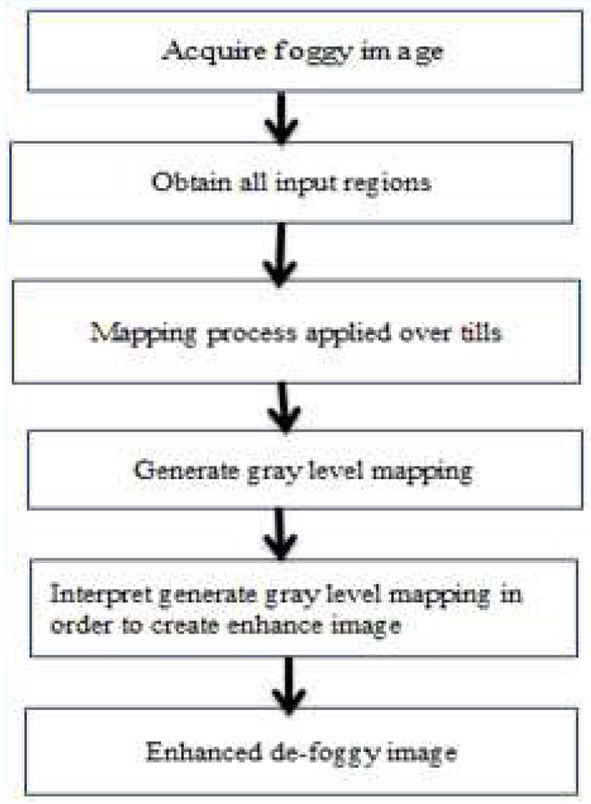
CLAHE procedure.

Histogram equalization is a technique for balancing histograms by increasing the contrast of the image by evenly dispersing gray values. In general, histogram equalization improves the images perceived quality and is advantageous when the image is intended for human viewing (13). By transforming gray values to a close approximation of a uniform distribution, histogram equalization improves contrast. However, unless all of the pixels in the input have the same gray level, the image in the output is scattered across various degrees of gray, resulting in gaps in the final histogram. Adaptive histogram equalization (AHE) is a modified kind of histogram equalization. In this method, the enhancement function is applied to all nearby pixels, and the transformation function is formed. This varies for AHE due to its contrast limiting. In Figure 3, this disadvantage is overcome by reducing the over-amplification of noise caused by the AHE approach. The estimated parameter contrast yields more efficient outcomes for CLAHE than AHE. As a result, when it comes to practice, the CLAHE technique outperforms the AHE technique (16). This is shown in Figure 3 (17).

### 3.2. Data augmentation

Data augmentation is the process of generating artificial increase of similar data such as transformations applied to the original data (18). Deep learning algorithms now achieve superior prediction results compared to other learning algorithms in terms of complexity and accuracy. However, in order to learn different parameters and properties, the model requires a large amount of data. Obtaining a vast amount of data in a medical center is usually difficult, so, by employing augmentation approaches, we can simply obtain the required amount of data.

Our dataset was gathered from several hospitals and EQA centers before being taken by smartphone in a regional lab and labeled by professionals. There are four (named) classes available: thin, thick, clean, and dirty. In each class, the amount of data is 175, 235, 300, and 182 for thin, thick, clean, and dirty, respectively. As a result, as we've seen in the literature, the performance of any method is influenced not only by the size of the dataset, but also by the unequal distribution of data within each class (19). The amount of data between thin and clean is vast and it almost has a relationship of clean being twice as large as thin in terms of data. As a result, we employed data augmentation to balance and increase the overall number of data in the random generation. The data augmentation algorithm is given as follows.

*Data Augmentation algorithm*

//Input: Enhanced image

//Output: Artificial image

Begin:

Assign RotationRange, WidthShiftRange,

HeightShiftRange, ShearRange,

ZoomRange;

SetHorizontalFlip(=true‘), FillMode(‘constant‘);

DataGen=(RotationRange, WidthShiftRange,

HeightShiftRange, ShearRange,

ZoomRange,

HorizontalFlip, FillMode;

Return DataGen;

End

### 3.3. Batch normalization

We applied a batch normalization before applying it to the next layer in the network to normalize the activations of the supplied input volume. While it helps minimize the number of epochs needed to train the neural network and stabilizes training, it slows down the training time of our network.

### 3.4. Feature extraction

Smear is evaluated according to the classified labels. Textural features of images can be utilized to precisely identify and classify sputum smear energy (depth). As we know, in CNN, the early convolutional layers extract texture at a high level rather than accounting for energy or texture depth. Using kernels and pooling, each convolutional layer generates higher level features from raw pixel inputs. Furthermore, because medical images are inherently complex, our dataset requires color modification rather than the RGB default color being used to gain better features. To examine the effect of color alterations performed within the HSV color module, the results are then fed to the SVM, CNN, and K-NN algorithms. To overcome the constraint of energy texture extraction, the GLCM handcrafted feature extractor was utilized.

**Figure 4:**
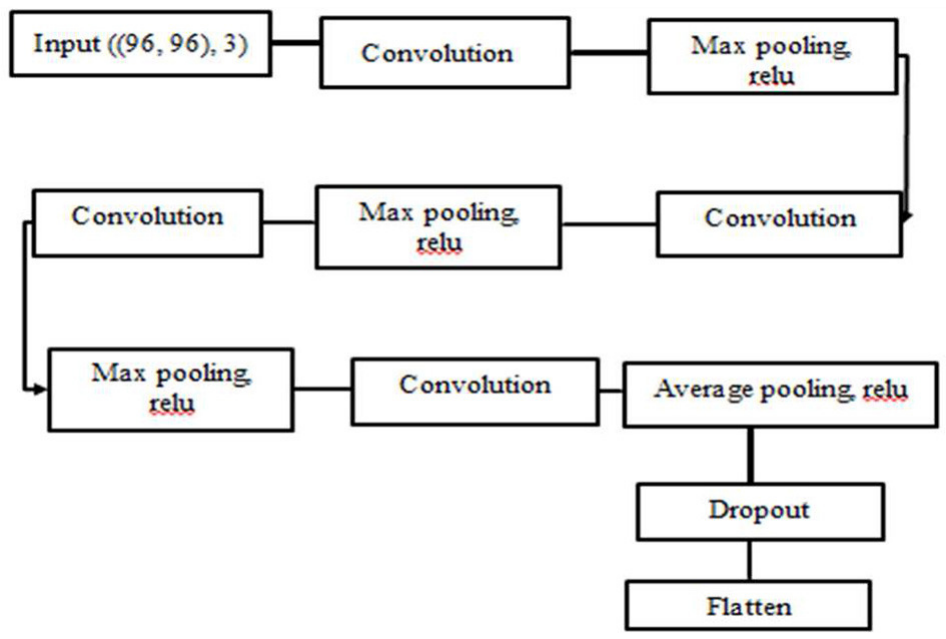
CNN feature extraction architecture.

CNN was employed as a feature extractor algorithm. Because CNN has a large impact on image analysis, the images passed through many layers of the hidden layers, and important information were learnt for the recognition phase by applying operations to the image such as convolution and pooling (20). The ReLU activation function was chosen since it outperforms the other activation functions in our model. Max pooling was also used since it reduces the size of the function vector and is appropriate for noisy images. The architecture of our network is depicted in Figure 4.

### 3.5. Classification algorithm

Image classification has arisen as a crucial subject in the field of computer vision. Object categorization is a relatively simple operation for humans, but it has proven to be a serious difficulty for computers. Image classification is the process of categorizing images into one of several predefined classifications. We used the following methods as classifiers in our model based on the current state of the art. For feature extraction, we use slow learners rather than deep classification algorithms like CNN. As mentioned, there are various slow learner categorization approaches. The KNN and SVM classifiers from those methods were used. The KNN method is simple to apply and has been used to solve classification and regression problems with multi-class labels. Furthermore, experts recommend employing deep learning classifiers such as CNN when there is a large amount of data and processing time is not an issue. However, it was difficult to collect sufficient data from the health centers, also computer resources were limited, and because computing time was an issue, we discovered that using slow learners was useful.

When it comes to slow learner classification algorithms, both SVM and KNN operate extremely well and effectively in both binary and multi-classifier classification problems by adding kernel function in SVM and K value in KNN (21). However, SVMs performance is classified differently in binary and multiclass, in addition to requiring extra work to make the SVM algorithm's kernel operate. This indicates that the SVM's performance declines in multiclass classification, even if it can be implemented. Deep learning is the alternative classifier for slow learners which operates on the basis of probability. As a result, despite its ease of construction, the rate of misclassification is substantial unless RF classifiers are utilized. K-Nearest Neighbors, or KNN for short, is a supervised machine learning technique that uses a labeled (Target Variable) dataset to predict the class of a new data point. The KNN approach is a reliable classifier that is frequently used as a benchmark for more complicated classifiers like artificial neural network (ANN) and support vector machine (SVM).

## 4. Results and discussion

### 4.1. Experimental setup

The models were built with Keras (Tensor Flow as a backend) and the anaconda spyder editor, as well as an HP Desktop computer with an Intel(R) Core (TM) i7-8700U CPU @ 3.19GHz and 8.00 GB RAM, Tensor Flow (22), Keras (23), pillow, and OpenCV libraries, all written in Python 3.9.

### 4.2. Dataset

A digital camera (Camon 15) was used in this study to collect sputum smear slide images which were labeled by TB laboratory experts. Thin, thick, clean, and dirty images are gathered during the data gathering procedure. Image augmentation was used on the improved datasets to balance the number of data in each class and also to improve the classification's accuracy model. The images are all jpgs with a resolution of 96 × 96 pixels. There are 8,000 images in the dataset fed into the feature extractor, with 2,000 images in each class. Our data was divided into two categories: training and testing, with 75:25 ratios of 6,000 for training the model and 2,000 for testing the models performance to determine how well it generalizes. The dataset description is presented in Table 1.

**Table 1:** Dataset description.

**Label**	**Resolution**	**Extension**	**Quantity**
Clean	96 × 96	JPG	2,000
Dirty	96 × 96	JPG	2,000
Thin	96 × 96	JPG	2,000
Thick	96 × 96	JPG	2,000

### 4.3. Experimental results

We were able to optimize several experiments using the proposed end to end CNN feature extractor and classifier using softMax through testing various optimization algorithms, learning rates, dropout layer, activation functions, and the number of epochs to be used to increase the accuracy rates and minimize the cost function of the models. We used a manually constructed feature builder and a slow learner algorithm after seeing the consequences of the other experimented methods. Our primary goals was to achieve suitable results while utilizing a considerable amount of processing time and computation resources. The results achieved show that both the GLCM feature extractor and the deep learning feature extractor performed well on their own and in combination with the CNN, KNN, and SVM classifiers.

#### 4.3.1. SVM classification using CNN features

The findings of our inquiry may have been either polynomial or RBF, as shown in Table 2. However, based on the results of our experiments, polynomial is the preferred choice for the present study. As a result, we used this method in the SVM multiclass classifier using a kernel function. The SVM approach provides polynomial, RBF, sigmoid, and linear kernel functions. The grid search method employed optimal parameters. Table 2 presents the results achieved using the SVM parameters such as accuracy, recall, precision, and F1-score.

**Table 2:** Results of SVM using CNN feature **s**.

**SVM using CNN features**	**Precision**	**Recall**	**F1-score**
Clean	0.71	0.49	0.58
Dirty	0.68	0.63	0.65
Thin	0.79	0.96	0.87
Thick	0.82	0.96	0.89
Average accuracy	0.759		

**Figure 5:**
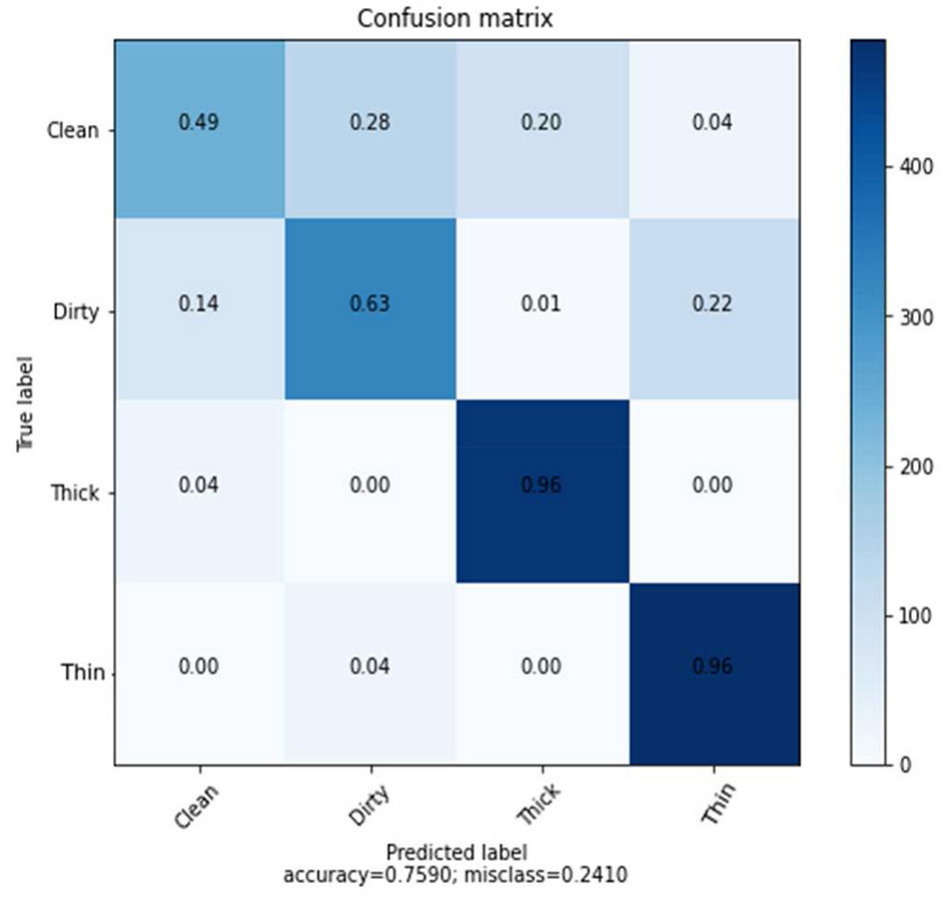
Confusion matrix of SVM using CNN features.

The confusion matrix shown in Figure 5 presents the prediction rate of each class and loss (miss classification) rate.

The result is based on 8,000 samples from our dataset, of which 6,000 were used for training and 2,000 for testing the performance of the model with 128 dimensions. The clean prediction rate is 49%, which is the lowest when compared to the other classifications, which are 63, 96, and 96% for dirty, thick, and thin, respectively. Within the CNN feature and SVM classifier, the average accuracy achieved is 75.90%.

#### 4.3.2. SVM classification using GLCM features

Success was achieved using the GLCM handcrafted texture-based feature extraction approach whose results were fed into the SVM classifier. Table 3 presents the precision, accuracy, recall, and F1-score achieved.

**Table 3:** Results of SVM using GLCM feature**s**.

**SVM using GLCM features**	**Precision**	**Recall**	**F1-score**
Clean	0.48	0.16	0.24
Dirty	0.62	0.58	0.60
Thin	0.77	0.94	0.84
Thick	0.61	0.91	0.73
Macro avg.	0.62	0.65	0.60
Weighted avg.	0.62	0.65	0.61
Average accuracy	0.651		

**Figure 6:**
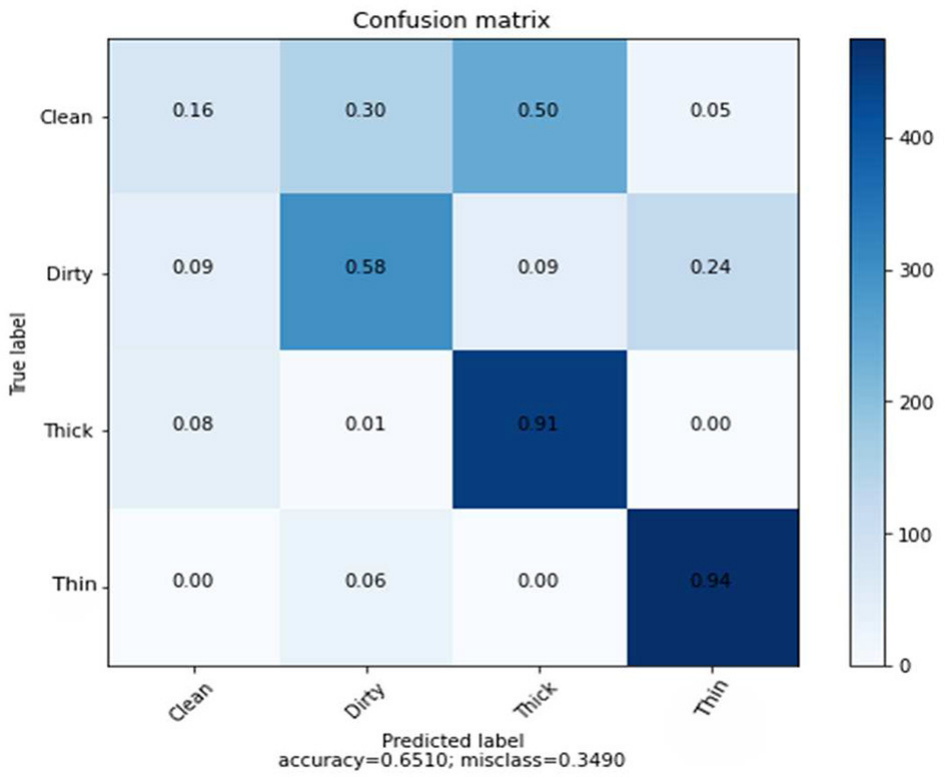
Confusion matrix of GLCM features using SVM classifier.

As demonstrated in Table 3, the number of characteristics in this experiment is small, and the classification algorithm achieves 65% average accuracy. Figure 6 depicts the rate of the recognition of each class.

We discovered through experiments using the GLCM, CNN, and combined features that deep learning features have a higher prediction rate than shallow learning features, and that combined features outperform both deep learning and handcrafted features.

#### 4.3.3. SVM classification using a combination of GLCM and CNN features

The CNN provides 128 features to the SVM classifier, and a 5-dimensional GLCM was combined from 6,000 training samples and 2,000 randomly chosen test samples. Hybrid features enable SVM classifier to achieve an average accuracy of 85%. Table 4 shows the results of the precision, recall, and F1-score.

**Table 4:** Precision, recall and F1-score of combined features using SVM.

**Combined features using SVM**	**Precision**	**Recall**	**F1-score**
Clean	0.90	0.84	0.87
Dirty	0.87	0.63	0.73
Thin	0.97	0.92	0.94
Thick	0.72	1.00	0.83
Macro avg.	0.86	0.85	0.84
Weighted avg.	0.86	0.85	0.84
Accuracy avg.	0.8455		

**Figure 7:**
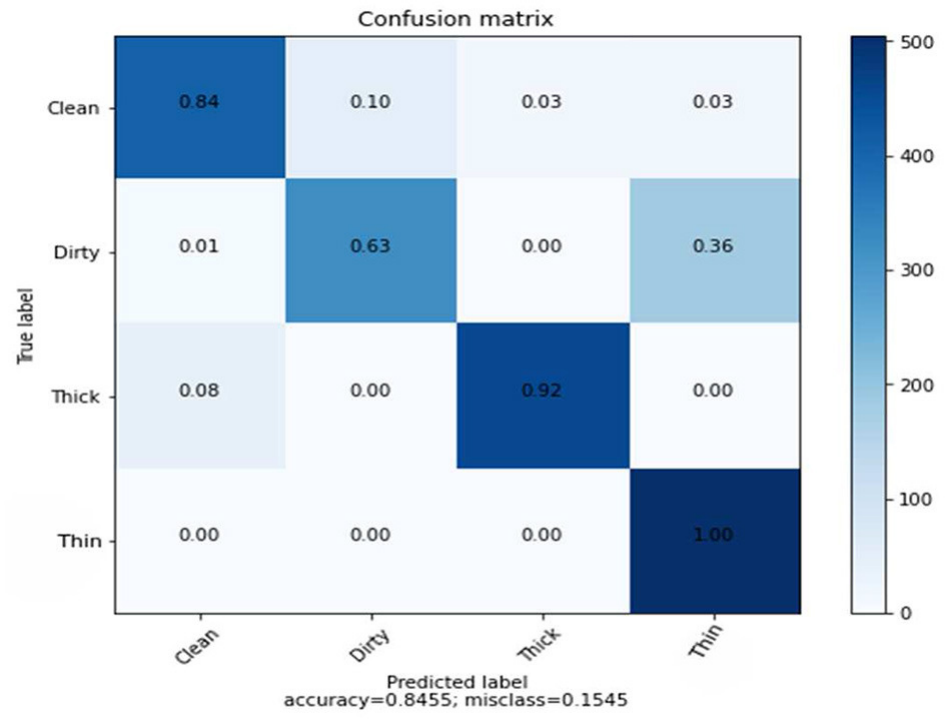
Confusion matrix of combined features using SVM.

The results of the three tests, which include GLCM, SVM, and combined features of GLCM and SVM, and SVM utilizing CNN feature, differ for each class. Figure 7 indicates the counts from predicted and actual values between the true and predicted class belonging to each class using a confusion matrix.

#### 4.3.4. KNN classification using GLCM features

The KNN algorithm feeds GLCM features with five dimensions into 6,000 samples for model training and 2,000 samples for model testing in order to make predictions. Table 5 shows the results obtained for precision, recall, and F1-Score with average accuracy.

**Table 5:** Result of KNN classification using GLCM features.

**KNN using GLCM features**	**Precision**	**Recall**	**F1-score**
Clean	0.76	0.76	0.76
Dirty	0.83	0.80	0.81
Thin	0.97	0.97	0.97
Thick	0.91	0.95	0.93
Macro avg.	0.87	0.87	0.87
Weighted avg.	0.87	0.87	0.87
Average accuracy	0.8695		

**Figure 8:**
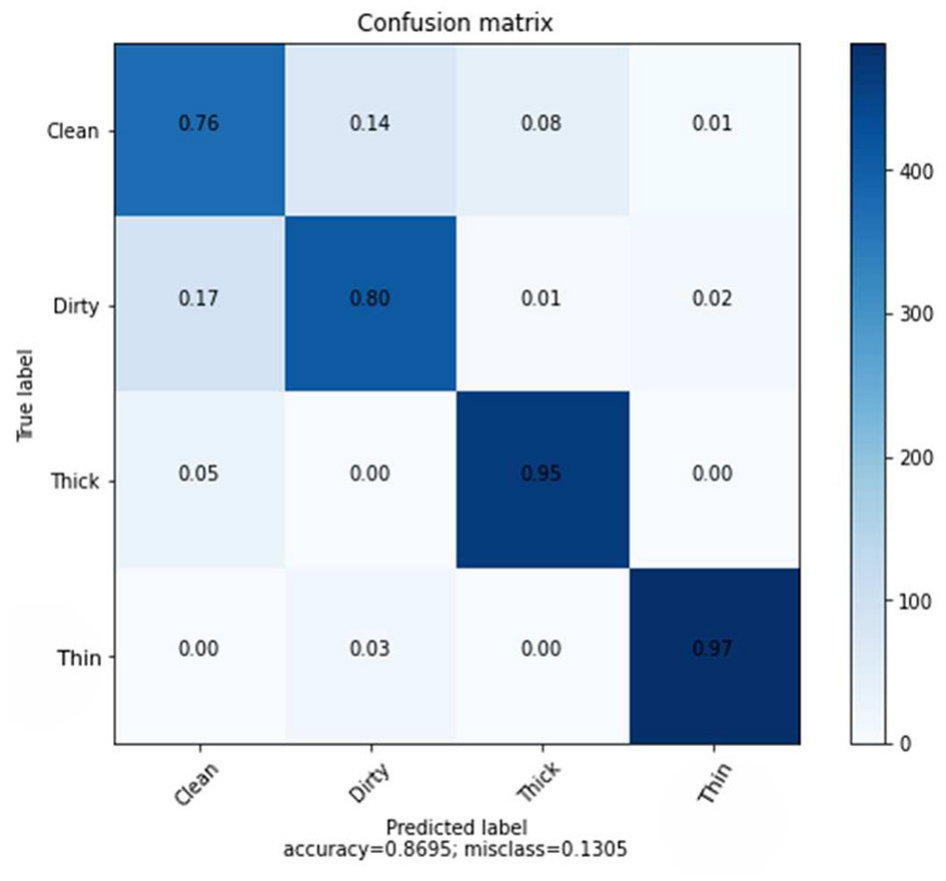
Confusion matrix of GLCM features using KNN.

The average accuracy of KNN classifiers using Hand-crafted features of GLCM is lower than that of deep learning, as shown in Figure 8. Figure 8 shows the results of the prediction rate as a function of loss.

The clean class received a score of 76%, followed by dirty, thick, and thin with scores of 80, 95, and 97% respectively. Furthermore, the overall average accuracy is 86.95%, as displayed in Figure 8 and Table 5. The relative miss classification rates for the classes clean, dirty, thick, and thin are 24, 20, 5, and 3%, respectively. Clean has a miss classification rate that is six times higher than thin.

#### 4.3.5. KNN classification using CNN features

In this experiment, a KNN algorithm with a K-value of 7 and an ideal parameter for Euclidean distance were used. KNN classifier with 128 dimensions and 6,000 training samples and 2,000 test samples achieved an average accuracy of 93%. Table 6 shows the precision, recall, and F1-score results for each class.

**Table 6:** Result of KNN classification using CNN features.

**KNN using CNN features**	**Precision**	**Recall**	**F1-score**
Clean	0.91	0.85	0.88
Dirty	0.92	0.95	0.93
Thin	0.95	1.00	0.98
Thick	0.94	0.94	0.94
Macro avg.	0.93	0.93	0.93
Weighted avg.	0.93	0.93	0.93
Average accuracy	0.9325		

**Figure 9:**
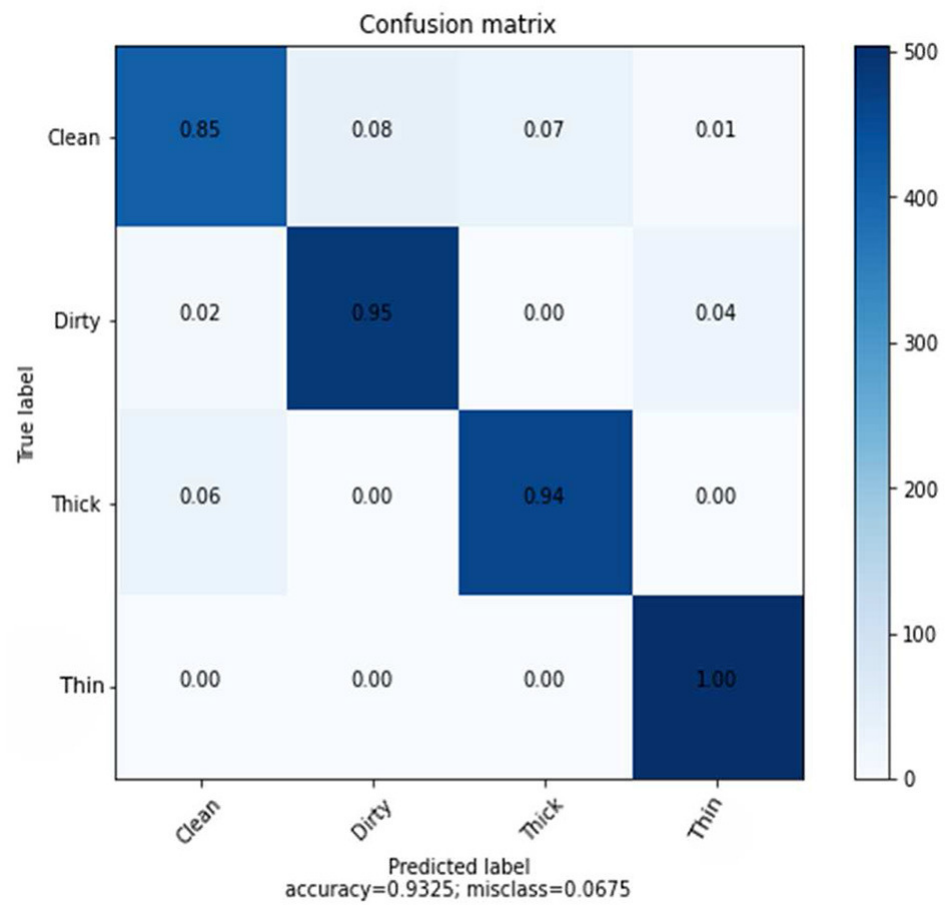
Confusion matrix KNN using CNN feature.

The experimental result in the recall matrix for the class thin is completely accurate, as can be seen in Table 6. The prediction rate for each class using the deep feature in comparison to the KNN method is shown in Figure 9.

#### 4.3.6. KNN classification using a combination of GLCM and CNN features

The combined features of GLCM and CNN, which have a total dimension of 133 and are derived from a dataset of 6,000 training samples and 2,000 testing samples, also use the KNN classifier. Table 7 clearly displays the results of the precision, recall, and F1-score rate of the KNN algorithm using hybrid features.

**Table 7:** Precision, recall, F1-score of combined features using KNN.

	**Precision**	**Recall**	**F1-score**
Clean	0.90	0.95	0.92
Dirty	0.98	0.88	0.93
Thin	0.97	0.93	0.95
Thick	0.92	1.00	0.96
Macro avg.	0.94	0.94	0.94
Weighted avg.	0.94	0.94	0.94
Accuracy avg.	0.939		

**Figure 10:**
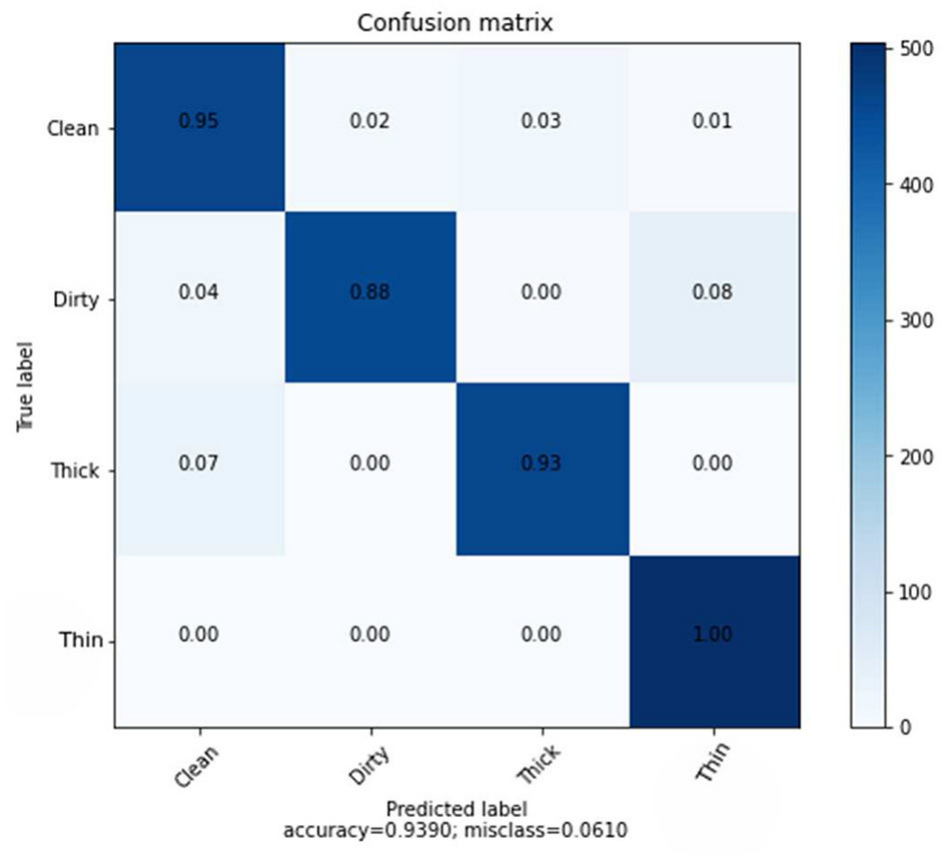
Confusion matrix of combined features using KNN.

Figure 10 provides specific data on the true level vs. prediction rate for each class. With the help of these combined characteristics and the KNN classifier, we were able to get the highest recognition rates for clean, dirty, thick, and thin, which are 95, 88, 93, and 100%, respectively. We performed six experiments, not including the ones we ran to determine the best parameter values.

**Figure 11:**
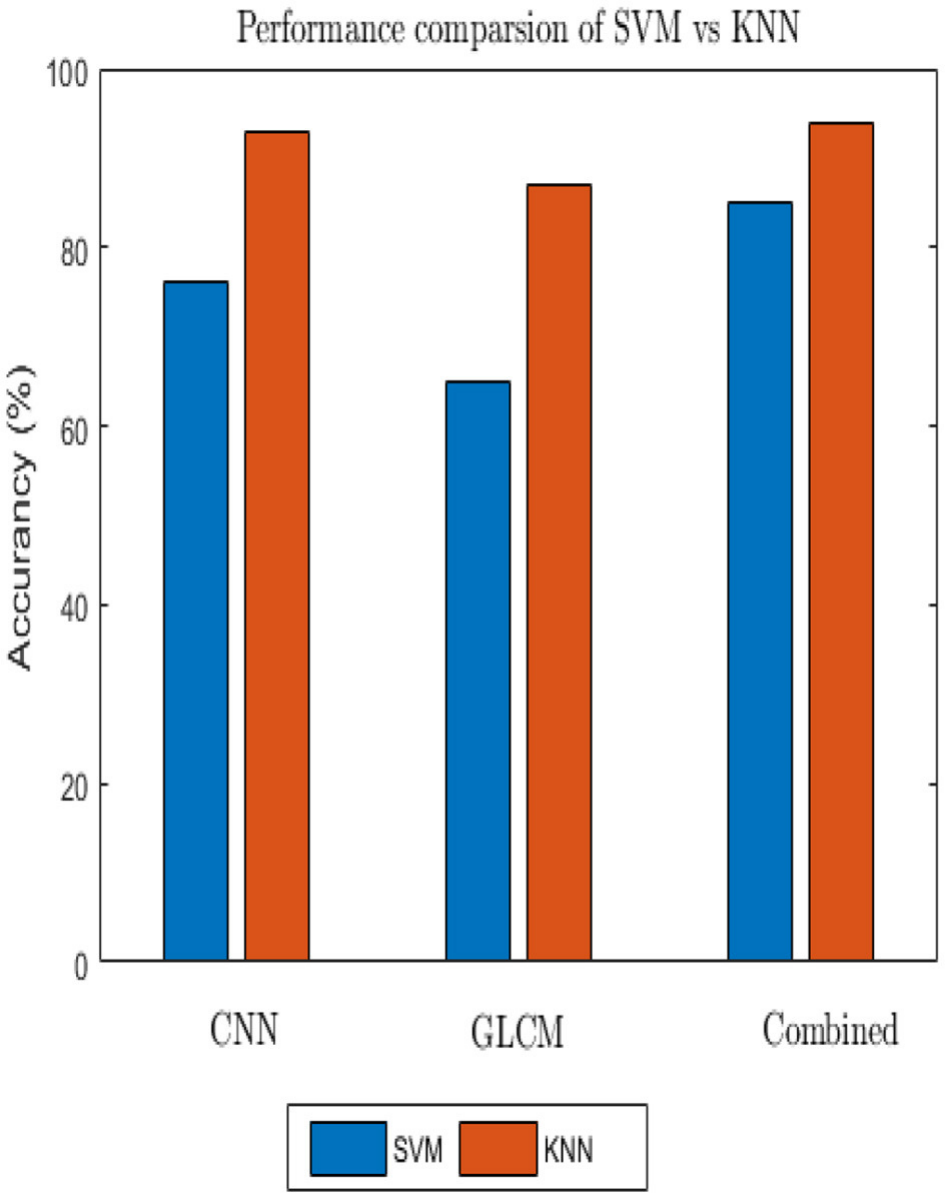
Comparison of SVM and KNN classifiers.

The accuracy the deep learning method on a slow learning classifier algorithm is superior to that of a professional feature method, according to all experiments conducted. Additionally, a hybrid is preferable to a single method from the results achieved. Figure 11 shows the performance of the SVM and KNN classification algorithms with three distinct features: GLCM, CNN, and a mix of GLCM and CNN.

## 5. Discussion

There are two distinct backgrounds in our situation. Bicubic interpolation was used to separate foreground and background after resampling in order to speed up computation and conserve time. The foreground form was divided using thresholding-based segmentation techniques. Then the Gaussian filter was applied because of handshaking, shadowing, and blur noise. The Gaussian filter, however, omits edge and detailed information. Due to its ability to maintain edges while collecting all available information, bilateral filtering techniques were used to resolve this problem. However, these techniques have shortcomings, including processing time and the insertion of additional artifacts. Then, other algorithms, like Gabor filters, were used which were helpful for images with complete information and textures. They are also good at maintaining edges, which helps the subsequent stage of feature extraction (24). It has been tested how various pre-processing techniques perform using the end-to-end CNN feature extraction and classification process. After applying CLAHE and successively feeding the output of the Gaussian filter into the input of the Gabor filter, a plausible output was achieved. We immediately observed how optimizers, learning rate, dropout, and activation function affects the deep learning classifier after selecting the pre-processing method. 84.41% training accuracy and 84.11% testing accuracy were finally recorded by 0.001 learning rate, 32 batch size, Adam optimizer, 0.25 dropout, and ReLU activation after numerous trials and errors. In addition, the SVM and KNN classifiers evaluated using the CNN features, achieved prediction rates of 76 and 93%, respectively.

Generally, employing a slow learning algorithm provides advantages in computational resources as well as prediction rate as far as robust features were employed. Additionally, it is quite simple to obtain a comparison result because there are only a few parameter optimizations. The performance of one algorithm varies from another, so choosing a promising method also has other problems because every algorithm has its own assumptions. According to various academic studies, SVM classifiers perform better than various classifiers (27), but not for our dataset.

According to the findings of several researchers, the number of classes has an impact on the classifier's effectiveness, with the prediction rate declining as the number of classes' increases as a result of developments in the binary class. Due to this, it performs worse than the KNN classifier. The KNN classifier, on the other hand, was created for multiclass issues, therefore connecting it with the kernel function doesn't require additional work. In conclusion, as each method was examined on a different set of assumptions, the accuracy achieved on ensemble feature vectors is higher than the accuracy reached by each individual because the limitations of CNN with GLCM in texture feature is concealed (28).

### 5.1. Comparison of proposed method with to previous methods

In order to compare the results of our proposed method, we choose prior studies that presented the performance of their proposed methods using MC and/or SZ datasets. Table 8 presents a contrast of the performance of the proposed method using our dataset with previous research for TB diagnosis, which used MC and/or SZ datasets' CXR images. The results show that the proposed method performed better than existing methods by 1.48%.

**Table 8:** Comparison of proposed method with existing works.

**References**	**Dataset**	**Accuracy (%)**
Asrat et al. (8)	–	82.7
Mekonen et al. (10)	–	72.7
Jaeger et al. (25)	SZ dataset	90.0
Lopes and Vailiati (26)	SZ and MC datasets	92.6
This study	Our dataset	94.0

## 6. Conclusion

This study showed that sputum smear quality inspection needs improvement but has received little attention despite its difficulties. Additionally, we discovered that a variety of methods, including image processing, machine learning, and deep learning techniques, are frequently used for the automatic identification of tuberculosis (TB) and other diseases. The aforementioned issues were resolved in this work by adding extra components like the integrated feature of GLCM employing CNN and the KNN classification approach. Once established, there will be less reliance on the knowledge of human specialists. To evaluate the caliber of sputum smears, KNN performed feature analysis using GLCM and CNN. The significance of the proposed system changed the inspection's accuracy and computation speed. The model has a 94% accuracy rating. As a result, our model addressed issues that the field has faced in the past, such as its reliance on specialized knowledge and inspection delays. Our model generates a range of results when run at different intervals. Although each iteration of SVM produces a different result, it didn't outperformed KNN or the other methods. To make the model more reliable and retain all the information, we plan to use deep learning segmentation techniques in the future.

## Data availability statement

The data analyzed in this study is subject to the following licenses/restrictions: The data that support the findings of this study are available on request from the corresponding author. The data are not publicly available due to privacy or ethical restrictions. Requests to access these datasets should be directed to ayodejisalau98@gmail.com.

## Author contributions

All authors listed have made a substantial, direct, and intellectual contribution to the work and approved it for publication.
